# Phytochemical Profiling by UHPLC–Q-TOF/MS and Chemopreventive Effect of Aqueous Extract of *Moringa oleifera* Leaves and Benzyl Isothiocyanate on Murine Mammary Carcinogenesis

**DOI:** 10.3390/molecules29061380

**Published:** 2024-03-20

**Authors:** Juan Pedro Rojas-Armas, Miriam Palomino-Pacheco, Jorge Luis Arroyo-Acevedo, José Manuel Ortiz-Sánchez, Hugo Jesús Justil-Guerrero, Jaime Teodocio Martínez-Heredia, Américo Castro-Luna, Crescencio Rodríguez Flores, Aldo Javier Guzmán Duxtan

**Affiliations:** 1 Laboratory of Pharmacology, Faculty of Medicine, Universidad Nacional Mayor de San Marcos, Lima 15001, Peru; jarroyoa@unmsm.edu.pe (J.L.A.-A.); hjustilg@unmsm.edu.pe (H.J.J.-G.); jmartinezh@unmsm.edu.pe (J.T.M.-H.); 2 Laboratory of Biochemistry, Faculty of Medicine, Universidad Nacional Mayor de San Marcos, Lima 15001, Peru; mpalominop1@unmsm.edu.pe; 3 Laboratory of Physiology, Faculty of Medicine, Universidad Nacional Mayor de San Marcos, Lima 15001, Peru; jortizs@unmsm.edu.pe; 4 Research Institute for Pharmaceutical Sciences and Natural Resources, Faculty of Pharmacy and Biochemistry, Universidad Nacional Mayor de San Marcos, Lima 15001, Peru; acastrol@unmsm.edu.pe; 5 Bruker Mexicana, Damas 130 Int.501 Col, San José Insurgentes, Mexico City 03900, Mexico; crescencio.rodriguez@bruker.com; 6 Department of Physical Chemistry, Faculty of Chemistry and Chemical Engineering, Universidad Nacional Mayor de San Marcos, Lima 15001, Peru; aldo.guzman@unmsm.edu.pe

**Keywords:** *Moringa oleifera*, benzyl isothiocyanate, cancer, breast, extract, rats

## Abstract

*Moringa oleifera* Lam, commonly known as moringa, is a plant widely used both as a human food and for medicinal purposes around the world. This research aimed to evaluate the efficacy of the aqueous extract of *Moringa oleifera* leaves (MoAE) and benzyl isothiocyanate (BIT) in rats with induced breast cancer. Cancer was induced with 7,12-dimethylbenz[a]anthracene (DMBA) at a dose of 60 mg/kg by orogastric gavage once only. Forty-eight rats were randomly assigned to eight groups, each consisting of six individuals. The control group (healthy) was called Group I. Group II received DMBA plus saline. In addition to DMBA, Groups III, IV, and V received MoAE at 100, 250, and 500 mg/kg/day, respectively, while Groups VI, VII, and VIII received BIT at 5, 10, and 20 mg/kg/day, respectively. Treatment was carried out for 13 weeks. Secondary metabolite analysis results identified predominantly quercetin, caffeoylquinic acid, neochlorogenic acid, vitexin, and kaempferol, as well as tropone, betaine, loliolide, and vitexin. The administration of MoAE at a dose of 500 mg/kg and BIT at 20 mg/kg exhibited a notable decrease in both the total tumor count and the cumulative tumor weight, along with a delay in their onset. Furthermore, they improved the histological grade. A significant decrease in serum levels of VEGF and IL-1β levels was observed (*p* < 0.001) with a better effect demonstrated with MoAE at 500 mg/kg and BIT at 20 mg/kg. In conclusion, this study suggests that both the aqueous extract of *Moringa oleifera* leaves and the benzyl isothiocyanate possess antitumor properties against mammary carcinogenesis, and this effect could be due, at least in part, to the flavonoids and isothiocyanates present in the extract.

## 1. Introduction

Breast cancer represents a significant challenge due to its high incidence and mortality rates in women worldwide [1]. According to data from the World Health Organization, in 2020, breast cancer was diagnosed in 2.3 million women and 685,000 women succumbed to the disease [2]. This scenario includes the marked adverse effects associated with chemotherapeutic agents [3]. Additionally, tumor cells have developed various mechanisms to induce resistance to chemotherapeutic treatments, a phenomenon known as chemoresistance, which significantly weakens efforts to combat breast cancer [4].

Natural compounds, through various mechanisms of action, present a potential inhibitor of drug resistance in cancer [5]. Furthermore, a synergistic interaction has been documented between secondary metabolites from plants and conventional chemotherapeutic drugs, which generates a greater antitumor effect and reduces associated toxicity [6]. In addition, several natural compounds have been identified for their chemosensitizing effect on cancer cells, increasing the cytotoxicity of drugs commonly used in chemotherapy [7].

*Moringa oleifera* Lam, which belongs to the Brassicales order, is widely known as moringa, horseradish tree, and drumstick tree, among other appellations, and is currently grown in various parts of the world [8]. It stands out as the most known and widely used among the 14 species recognized within the Moringa genus [9]. This plant is used both as food for human consumption and for medicinal purposes around the world, as numerous studies have identified various health benefits, including nutritional benefits and medicinal properties [10]. Its global recognition is due to its multiple medicinal applications, which has earned it the nickname “miracle tree” [11].

Scientific studies have shown the ability of *Moringa oleifera* to fight different types of cancer. The ethanolic and aqueous extracts of leaves has been reported to possess antiproliferative properties in colon cancer cell lines [12,13]. The hydroalcoholic extract of the leaves has shown an impact on Ehrlich solid tumor generated by the implantation of Ehrlich ascites carcinoma cells in mice, reducing the formation of micronuclei and DNA damage, and inhibiting the expression of the inducible nitric oxide synthase (iNOS), vascular endothelial growth factor (VEGF), p53 protein (p53) mutation, and B cell lymphoma 2 (Bcl-2) genes [14]. On the other hand, the aqueous extract of leaves has exhibited antiproliferative properties in A549 lung cancer cells, evidencing a pro-apoptotic action by significantly increasing the expression of p53, caspase-9, caspase-3, and caspase-7 proteins [15]. Furthermore, the methanolic extract of the leaves has significantly inhibited cell survival and induced apoptosis, associated with a marked increase in Bax and a decrease in Bcl-2 in the human prostate cancer cell line DU145 [16]. Similarly, inhibition of MCF-7 cell proliferation in breast cancer has been demonstrated by aqueous seed extract and the dichloromethane extract of moringa leaves [17,18].

The predominant phytochemical compounds in *Moringa oleifera* Lam are glucosinolates, followed by phenols and flavonoids. Leaves and seeds have been reported to harbor the highest concentrations of glucosinolates, which are precursors to isothiocyanates. Among them, benzylglucosinolate is the most abundant in leaves and, by the catalytic action of myrosinase, it is transformed into benzyl isothiocyanate [19,20].

The lack of specificity, high toxicity, and common resistance associated with conventional chemotherapy motivate the exploration of alternative sources with a more favorable profile in terms of efficacy and safety. Considering this context and taking into account that so far only in vitro antiproliferative activity of moringa leaf extracts and its main secondary metabolite, benzyl isothiocyanate, have been reported against breast cancer cells, with no in vivo studies available, we designed this research to evaluate the impact of MoAE and BIT on murine breast carcinogenesis.

## 2. Results and Discussion

### 2.1. Phytochemical Analysis of MoAE

**Figure 1:**
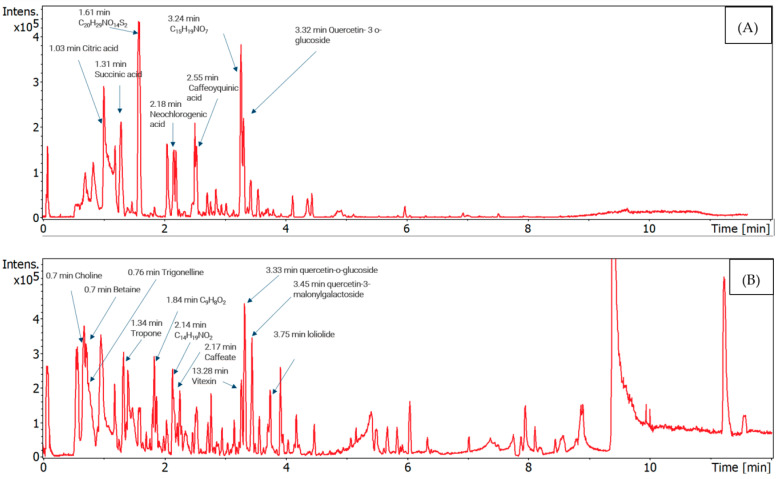
Chromatogram of the aqueous extract of moringa leaves, using UHPLC–Q-TOF/MS. (**A**) Negative ionization mode; (**B**) positive ionization mode.

The components identified in the MoAE by an ultra-performance liquid chromatography system coupled to a quadrupole time-of-flight mass spectrometer (UHPLC–Q-TOF/MS), either in negative or positive ionization mode, are presented in Figure 1, Table 1 and Table 2, respectively. In the negative mode, among the secondary metabolites, the presence of quercetin (glycosylated at C3) was determined, represented by quercetin-3-glucoside, quercetin-3-(6″-malonylglucoside) and quercetin-3-(6″-acetylglucoside). Other compounds such as caffeoylquinic acid, neochlorogenic acid, vitexin, and kaempferol 3-alpha-D-galactoside were also identified. Quinic acid, pinolenic acid, and rutin were detected with lower intensity.

In the positive mode, glycosylated quercetin, tropone, betaine, vitexin, loliolide, and kaempferol were identified. However, compounds such as ramelteon (TAK 375), D-pipecolic acid, isorhamnetin 3-glucoside, isoorientin, viscidulin I, 6-methylquinoline, 5-O-feruloylquinic acid, and rutin, among others, were detected with diminished intensity. In addition, primary metabolites such as sucrose, D-fructose; essential amino acids (which the organism cannot synthesize) leucine, isoleucine, phenylalanine, and valine; non-essential amino acids proline, arginine, glutamate and alanine; and the vitamins pyridoxine (vitamin B6), riboflavin (vitamin B2), and pantothenic acid (vitamin B5) were identified.

However, although authors such as Förster [19] and Waterman [20] recognize that BIT is a component of *Moringa oleifera* leaves, in this study its presence was not detected in MoAE. This may be attributed to our use of water extraction at 100 °C. Temperatures above 80 °C have been observed to lead to inactivation of myrosinase [20], thus preventing BIT formation. Therefore, the extraction of hot water at 100 °C poses a drawback, as it hinders the formation of BIT, a pharmacologically important compound. Al-Asmari [21] used gas chromatography coupled with mass spectrometry (GC/MS) to detect BIT in an alcoholic extract concentrated in a rotary evaporator at 50 °C, as reported.

**Table 1:** Chemical composition of aqueous extract of moringa analyzed by UHPLC–Q-TOF/MS (negative mode).

N°	Compound Name	Formula	Theoretical Mass (*m*/*z*)	Experimental Mass (*m*/*z*)	Error (ppm)	RT (min)	Ref.
1	Gluconic acid	C_6_H_12_O_7_	195.0510	195.0514	2.07	0.72	[22]
2	D-Arabinonic acid	C_5_H_10_O_6_	165.0405	165.0407	1.62	0.72	[22]
3	D-Fructose	C_6_H_12_O_6_	179.0561	179.0565	2.06	0.73	[23]
4	Threonic acid	C_4_H_8_O_5_	135.0299	135.0301	1.28	0.74	[24]
6	Quinic acid	C_7_H_12_O_6_	191.0561	191.0565	1.82	0.77	[25]
7	Sucrose	C_12_H_22_O_11_	341.1089	341.1096	1.95	0.78	[23]
8	Malic acid	C_4_H_6_O_5_	133.0142	133.0144	1.45	0.86	[22]
9	Malonic acid	C_3_H_4_O_4_	103.0037	103.0038	1.05	0.90	[26]
10	3-Aminobutanoic acid	C_4_H_9_NO_2_	102.0561	102.0561	0.67	0.96	[27]
11	Uric acid	C_5_H_4_N_4_O_3_	167.0211	167.0214	2.01	1.02	[22]
12	Citric acid	C_6_H_8_O7	191.0197	191.0202	2.59	1.03	[22]
13	Pyromucic acid	C_5_H_4_O_3_	111.0088	111.0089	1.55	1.03	[28]
14	Succinic acid	C_4_H_6_O_4_	117.0193	117.0195	1.18	1.31	[29]
15	L-Phenylalanine	C_9_H_11_NO_2_	164.0717	164.0720	2.00	1.86	[22]
16	2,5-dihydroxybenzoic acid	C_7_H_6_O_4_	153.0193	153.0196	1.62	2.09	[30]
17	Neochlorogenic acid	C_16_H_18_O_9_	353.0878	353.0886	2.16	2.18	[24]
18	Hydroxyphenyllactic acid	C_9_H_10_O_4_	181.0506	181.0510	1.98	2.27	[31]
19	2-Isopropylmalic acid	C_7_H_12_O_5_	175.0612	175.0616	2.07	2.50	[32]
20	Caffeoylquinic acid	C_16_H_18_O_9_	353.0878	353.0885	2.05	2.55	[33]
21	Saponarin	C_27_H_30_O_15_	593.1512	593.1523	1.78	2.94	[34]
22	Rutin	C_27_H_30_O_16_	609.1461	609.1469	1.28	3.19	[35]
23	Vitexin	C_21_H_20_O_10_	431.0984	431.0992	1.83	3.27	[32]
24	Quercetin 3-glucoside	C_21_H_20_O_12_	463.0882	463.0890	1.66	3.32	[35]
25	3-phenyllactic acid	C_9_H_10_O_3_	165.0557	165.0560	1.47	3.39	[31]
26	Quercetin 3-(6″-malonylglucoside)	C_24_H_22_O_15_	549.0886	549.0896	1.78	3.44	[35]
27	Quercetin 3-(6″-acetylglucoside)	C_23_H_22_O_13_	505.0988	505.0997	1.77	3.44	[31]
28	Kaempferol 3-alpha-D-galactoside	C_21_H_20_O_11_	447.0933	447.0941	1.87	3.56	[35]
29	Kaempherol 3-O-(6-malonylgalactopyranoside)	C_24_H_22_O_14_	533.0937	533.0946	1.63	3.72	[35]
30	Azelaic acid	C_9_H_16_O_4_	187.0976	187.0980	1.96	3.82	[31]
31	Hieracin	C_15_H_10_O_7_	301.0354	301.0361	2.27	4.37	[36]
32	Kaempferol	C_15_H_10_O_6_	285.0405	285.0411	2.17	4.87	[37]
33	Kaempferide	C_16_H_12_O_6_	299.0561	299.0571	3.40	4.93	[38]
34	6-Methoxyluteolin	C_16_H_12_O_7_	315.0510	315.0517	2.17	4.98	[39]
35	Pinolenic Acid	C_18_H_30_O_2_	277.2173	277.2178	1.64	8.54	[40]

**Table 2:** Chemical composition of aqueous extract of moringa analyzed by UHPLC–Q-TOF/MS (positive mode).

N°	Compound Name	Formula	Theoretical Mass (*m*/*z*)	Experimental Mass (*m*/*z*)	Error (ppm)	RT (min)	Ref.
1	D-Arginine	C_6_H_14_N_4_O_2_	175.1190	175.1186	2.01	0.66	[22]
2	Choline	C_5_H_13_NO	104.1070	104.1069	0.86	0.69	[31]
3	L-Glutamic acid	C_5_H_9_NO_4_	148.0604	148.0604	0.23	0.69	[22]
4	Betaine	C_5_H_11_NO_2_	118.0863	118.0861	1.31	0.70	[41]
5	Muramic acid	C_9_H_17_NO_7_	252.1078	252.1073	1.90	0.70	[42]
6	Glucosamine	C_6_H_13_NO_5_	180.0866	180.0863	1.94	0.71	[43]
7	D-Proline	C_5_H_9_NO_2_	116.0706	116.0705	0.90	0.75	[26]
8	Trigonelline	C_7_H_7_NO_2_	138.0550	138.0547	1.99	0.76	[44]
9	Furfural	C_5_H_4_O_2_	97.0284	97.0284	0.06	0.76	[45]
10	N2-Acetyl-L-ornithine	C_7_H_14_N_2_O_3_	175.1077	175.1079	1.03	0.8	[46]
11	Proline betaine	C_7_H_13_NO_2_	144.1019	144.1016	2.12	0.83	[47]
12	4-Hydroxypyridine	C_5_H_5_NO	96.0444	96.0444	0.10	0.87	
13	Glu Ala	C_8_H_14_N_2_O_5_	219.0975	219.0977	0.69	0.91	
14	L-Valine	C_5_H_11_NO_2_	118.0863	118.0861	1.31	0.93	[26]
15	D-Pipecolic acid	C_6_H_11_NO_2_	130.0863	130.0861	1.19	0.97	[48]
16	Isonicotinic acid	C_6_H_5_NO_2_	124.0393	124.0391	1.65	1.01	[31]
17	3-Aminosalicylic acid	C_7_H_7_NO_3_	154.0499	154.0496	1.75	1.15	[49]
18	D-Pyroglutamic acid	C_5_H_7_NO_3_	130.0499	130.0496	2.08	1.21	[50]
19	Pyridoxine (Vitamin B6)	C_8_H_11_NO_3_	170.0812	170.0812	0.18	1.21	[31]
20	Phenacylamine	C_8_H_9_NO	136.0757	136.0753	2.87	1.27	
21	Salsolinol	C_10_H_13_NO_2_	180.1019	180.1015	2.25	1.32	[51]
22	Tropone	C_7_H_6_O	107.0491	107.0490	1.32	1.34	[52]
23	Euparin	C_13_H_12_O_3_	217.0859	217.0855	1.94	1.34	[53]
24	2,6-Dihydroxynaphthalene	C_10_H_8_O_2_	161.0597	161.0594	1.90	1.34	
25	D-Isoleucine	C_6_H_13_NO_2_	132.1019	132.1017	1.55	1.39	[26]
26	Vidarabine	C_10_H_13_N_5_O_4_	268.1040	268.1035	1.98	1.39	[54]
27	N-(1-Deoxy-D-fructos-1-yl)-D-leucine	C_12_H_23_NO_7_	294.1547	294.1540	2.48	1.44	
28	L-Leucine	C_6_H_13_NO_2_	132.1019	132.1016	2.31	1.46	[26]
29	2-Pyrrolidinone	C_4_H_7_NO	86.0600	86.0599	1.63	1.48	[55]
30	Cryptochlorogenic acid	C_16_H_18_O_9_	355.1024	353.0882	1.10	1.69	[24]
31	Pantothenic acid	C_9_H_17_NO_5_	220.1179	220.1173	2.95	1.92	[32]
32	Caffeate	C_9_H_6_O_3_	163.0390	163.0385	2.89	2.17	[56]
33	(±)-Furaneol	C_6_H_8_O_3_	129.0546	129.0543	2.49	2.32	[57]
34	Chlorogenic acid	C_16_H_18_O_9_	355.1024	355.1016	2.14	2.53	[24]
35	Glu Phe	C_14_H_18_N_2_O_5_	295.1288	295.1293	1.53	2.55	[22]
36	6-Methylquinoline	C_10_H_9_N	144.0808	144.0804	2.61	2.74	[58]
37	Riboflavin (Vitamin B2)	C_17_H_20_N_4_O_6_	377.1456	377.1449	1.75	2.86	[32]
38	Coumarin	C_9_H_6_O_2_	147.0441	147.0438	2.08	2.87	[59]
39	Corchoionol C 9-glucoside	C_19_H_30_O_8_	387.2013	387.2003	2.70	2.90	
40	Saponarin	C_27_H_30_O_15_	595.1657	595.1649	1.42	2.94	[60]
41	Isoorientin	C_21_H_20_O_11_	449.1078	449.1072	1.42	2.96	[60]
42	5-O-Feruloylquinic acid	C_17_H_20_O_9_	369.1180	369.1174	1.65	3.07	[61]
43	Rutin	C_27_H_30_O_16_	611.1607	611.1592	2.39	3.20	[25]
44	Vitexin	C_21_H_20_O_10_	433.1129	433.1120	2.13	3.28	[32]
45	Quercetin 3-O-glucoside	C_21_H_20_O_12_	465.1028	465.1016	2.48	3.33	[25]
46	Quercetin	C_15_H_10_O_7_	303.0499	303.0492	2.50	3.54	[35]
47	Kaempferol 3-alpha-D-galactoside	C_21_H_20_O_11_	449.1078	449.1068	2.31	3.58	[35]
48	Isorhamnetin 3-glucoside	C_22_H_22_O_12_	479.1184	479.1173	2.30	3.64	[25]
49	Quercetin 3-(6″-malonylgalactoside)	C_24_H_22_O_15_	551.1031	551.1022	1.70	3.65	[35]
50	Viscidulin I	C_15_H_10_O_7_	303.0499	303.0493	2.08	3.65	[37]
51	Quercetin 3-(6″-acetylglucoside)	C_23_H_22_O_13_	507.1133	507.1124	1.81	3.68	[31]
52	Methyl cinnamate	C_10_H_10_O_2_	163.0754	163.0751	1.57	3.71	[62]
53	Kaempferol	C_15_H_10_O_6_	287.0550	287.0543	2.38	3.74	[37]
54	loliolide	C_11_H_16_O_3_	197.1172	197.1168	2.14	3.75	[63]
55	3,4-Dimethylstyrene	C_10_H_12_	133.1012	133.1008	2.83	3.77	
56	Isorhamnetin	C_16_H_12_O_7_	317.0656	317.0648	2.43	3.82	[61]
57	Cyanidin 3-(6″-acetylglucoside)	C_23_H_22_O_12_	491.1184	491.1176	1.64	4.00	[64]
58	Traumatic acid	C_12_H_20_O_4_	229.1434	229.1430	1.90	4.85	[65]
59	Dihydroactinidiolide	C_11_H_16_O_2_	181.1223	181.1218	2.79	5.50	[66]
60	2-Heptyl-4-hydroxyquinoline	C_16_H_21_NO_2_	260.1645	260.1638	2.71	5.93	
61	2-heptylquinolin-4(1H)-one	C_16_H_21_NO	244.1696	244.1689	2.83	6.05	
62	Quercetin tetramethyl (5,7,3′,4′) ether	C_19_H_18_O_7_	359.1125	359.1114	3.14	6.98	[67]
64	13E-Docosenamide	C_22_H_43_NO	338.3417	338.3408	2.78	9.29	[68]

### 2.2. Phenolic Content and Antioxidant Activity of MoAE

The total phenolic content of MoAE was quantified as 135.08 ± 0.64 mg equivalent gallic acid per gram of dry extract, using the equation y = 1241x + 0.1619 of the standard gallic acid curve. The antioxidant capacity, determined by the 2,2-Diphenyl-1-picrylhydrazyl (DPPH) radical scavenging assay, is shown in Table 3. Manguro and Lemmen [69], using spectroscopic methods to characterize phenols in the methanolic extract of *M. oleifera* leaves from Kenya, reported the presence of flavonol glycosides, kaempferol, syringic acid, gallic acid, rutin, and quercetin. On the other hand, Al-Asmari [21], using gas chromatographic and mass spectrometric methods, identified mainly thiocyanates in *M. oleifera* of Saudi Arabia and did not detect phenolic compounds and flavonoids.

In this investigation, the aqueous extract of *M. oleifera* showed antioxidant activity, consistent with the findings of the study of Peñalver [70], which linked a higher content of phenolic compounds with a greater antioxidant capacity. Similarly, Fitriana [71] demonstrated a potent free radical scavenging activity with an IC_50_ of 49.30 μg/mL in the DPPH assay using the methanolic extract of *Moringa oleifera* leaves, while we observed a value of 66.66 ± 0.53 μg/mL (Table 3). Additionally, a significant antioxidant potential of peptides derived from *M. oleifera* leaves has been reported [72].

Reactive oxygen species (ROS) play an important role in the modification of various cell signaling pathways that create an environment conducive to tumor development [73]. Furthermore, they have an impact on treatment response and the development of drug resistance [74]. This is due to the ability of free radicals to communicate within and outside of cells, serving as secondary messengers and regulating tumor cell signaling [75]. Studies have indicated that polyphenols, among other mechanisms, can influence these signaling pathways, exerting antitumor effects [76]. Considering the substantial oxidative stress observed both internally and externally in breast cancer cells [77], it is reasonable to assume that MoAE, with its antioxidant properties, may have a beneficial impact on this disease. This potential effect could be attributed to its identified components, such as quercetin, known for its potent antioxidant properties [78,79], as well as kaempferol [80].

### 2.3. MoAE Activity on Cancer Induced in Rats

**Figure 2:**
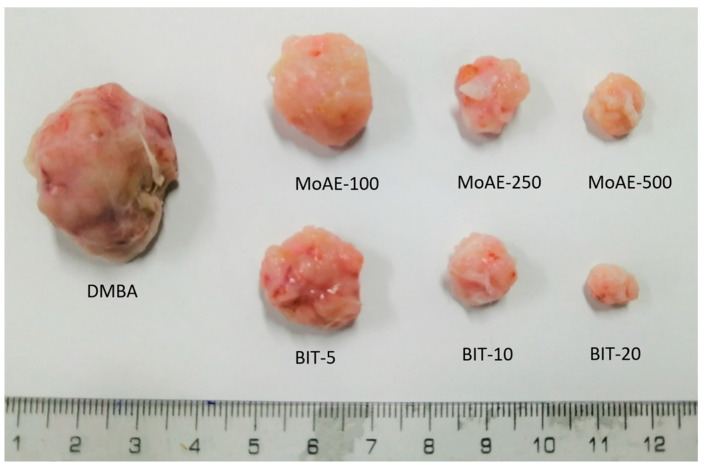
Photographs of tumors removed from DMBA-induced breast cancer rats treated for 13 weeks with MoAE and BIT.

The impact of treatment for 13 weeks with MoAE and BIT is presented in Table 4. MoAE at 500 mg/kg generated the best effect in reducing the total number of tumors, showing 10 tumors compared to 18 tumors in the cancer-induced BMBA group; this marked a reduction of 44% in the mean tumor count within this particular group. Similarly, BIT at a dose of 20 mg/kg reduced the average number of tumors by 33%. The delay in tumor onset was prolonged when MoAE at 500 mg/kg and BIT at 20 mg/kg were administered, recording durations of 67.80 ± 9.86 days and 71.75 ± 5.38 days, respectively. Furthermore, a more significant tumor size was observed in the DMBA group, and this size was reduced as a result of treatment (Figure 2), demonstrating a decrease in cumulative tumor weight with MoAE at 500 mg/kg and BIT at 20 mg/kg, representing 70.14% and 67.56%, respectively (Table 4).

**Figure 3:**
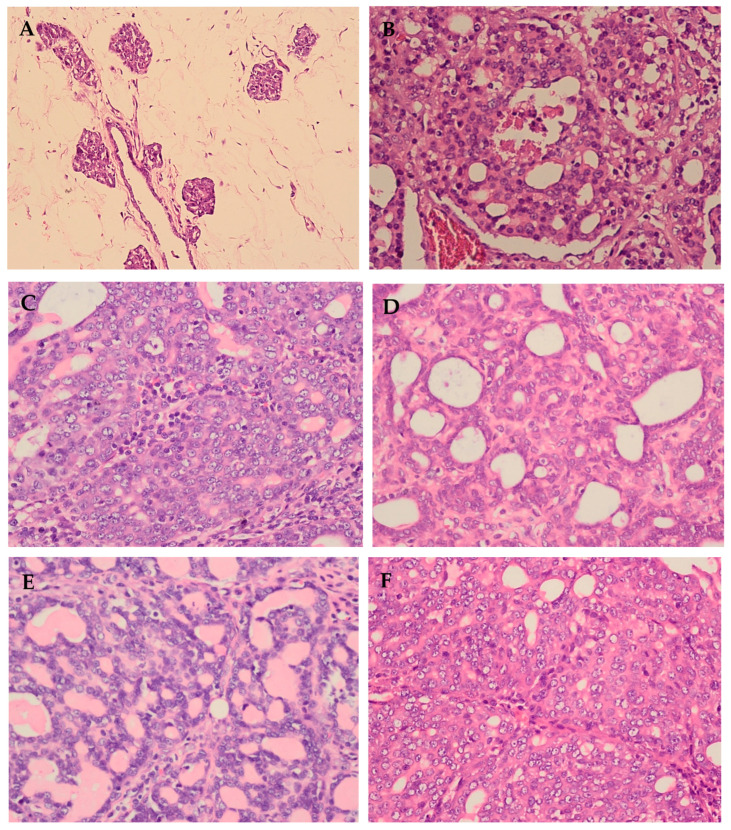

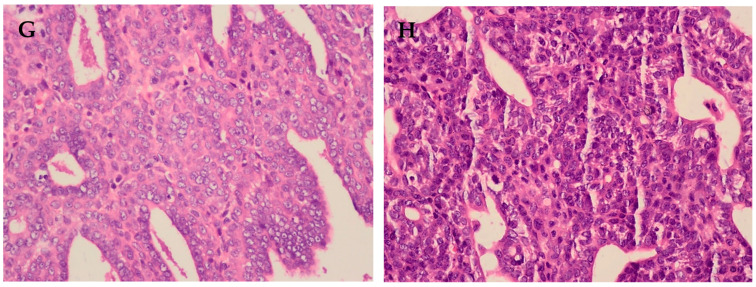
Microphotographs of breast cancer in rats induced by DMBA treated for 13 weeks with MoAE and BIT. (**A**) Normal, (**B**) DMBA, (**C**) DMBA + MoAE 100 mg/kg, (**D**) DMBA + MoAE 250 mg/kg, (**E**) DMBA + MoAE 500 mg/kg, (**F**) DMBA + BIT 5 mg/kg, (**G**) DMBA + BIT 10 mg/kg, (**H**) DMBA + BIT 20 mg/kg.

On histopathological examination, it was observed in the control group that the mammary gland is organized in clusters of small tubulo-alveolar glands called lobules, which are separated by an appreciable amount of lax stroma, draining their secretions into the lactiferous ducts. No nuclear pleomorphism or mitosis was observed (Figure 3A). In contrast, in the DMBA group, an epithelial neoplasm was observed with very little stroma, organized in solid areas with tubular formations and in the presence of nuclear pleomorphism (Figure 3B). In the group treated with moringa extract at 100 mg/kg, epithelial neoplasia with very little stroma was observed, organized predominantly in tubular formations with marked nuclear pleomorphism and mitosis (Figure 3C). In the moringa group at 250 mg/kg, epithelial neoplasia was also observed to be organized in solid areas with tubular formations and with the presence of moderate nuclear pleomorphism (Figure 3D). In contrast, in the moringa group at 500 mg/kg, epithelial neoplasia with scant stroma was observed, organized predominantly in tubular formations with the presence of nuclear pleomorphism (Figure 3E).

Regarding the group treated with benzyl isothiocyanate at 5 mg/kg, it presented a limited effect, since epithelial neoplasia with very little stroma was observed, organized predominantly in solid areas and some tubular formations, with marked nuclear pleomorphism and mitosis (Figure 3F). In the BIT group at 10 mg/kg, epithelial neoplasia was observed with a small stroma, organized in solid areas with some tubular formations, and with the presence of marked nuclear pleomorphism (Figure 3G). In the BIT group at 20 mg/kg, epithelial neoplasia with very little stroma was observed, organized with a predominance of solid areas and some tubular formations, with nuclear pleomorphism and mitosis (Figure 3H).

This analysis allowed determination of the histological grade, showing a positive effect of moringa extract treatment at 250 and 500 mg/kg, as well as benzyl isothiocyanate at 20 mg/kg, where the histological grade was I compared to grade II in the DMBA group (Table 5).

A beneficial treatment effect was observed in the DMBA-induced breast cancer model when using MoAE and BIT, especially at doses of 500 mg/kg and 20 mg/kg, respectively. In the macroscopic evaluation, a decrease in the number of tumors was evident, as well as in the total cumulative tumor weight, together with a delay in their appearance (see Table 4). During the histopathological examination, enhancements in tubular differentiation and nuclear polymorphism were observed, which positively influenced the histological grade (see Table 5). It is plausible that this antitumor effect of *Moringa oleifera* is associated, at least in part, with the presence of its phenolic components, including flavonoids. In this context, flavonoids have been shown to exhibit anticancer properties against breast cancer, being able to induce the expression of various tumor suppressor genes that contribute to mitigating cancer progression and metastasis [81]. Additionally, natural flavonoids have been reported to possess antioxidant, anti-inflammatory, and anticancer activities through various pathways. These compounds can inhibit cell proliferation, arrest the cell cycle by suppressing the NF-kB pathway in various types of cancer, and promote apoptosis in breast cancer [82].

Regarding quercetin, several studies, including those of a basic, epidemiological, and genetic nature, suggest the possibility of its contribution in the treatment of breast cancer [83]. For example, quercetin has been reported to cause a decrease in cell viability and cell cycle arrest in the G2/M phase, associated with a reduction in proteosomal enzyme activities [84]. Similarly, it has been found to inhibit tumor invasion by suppressing PKC delta/ERK/AP-1-dependent activation of matrix metalloproteinase MMP-9 in MCF-7 breast carcinoma cells [85]. Further experiments have revealed that quercetin inhibits the growth of the MCF-7 cancer cell line, induces apoptosis, and, in in vivo studies, reduces tumor volume in mice with tumors by CT-26 and MCF-7 cells, increasing animal survival [86]. In addition, it has been found to induce cytotoxicity in breast cancer cells, arrest cell cycle progression in the S phase, and induce tumor regression in mice [87]. In particular, quercetin, when encapsulated in lipid nanoparticles, intensifies its toxic effect on MCF-7 breast cancer cells [88].

In another perspective, *Moringa oleifera*-derived isothiocyanates have demonstrated a remarkable ability to inhibit viability in nine breast cancer cell lines [89]. Benzyl isothiocyanate (BIT) has shown efficacy in suppressing the growth of MDA-MB-231 and MCF-7 human breast cancer cells, with effects including cell cycle arrest and apoptosis induction [90]. In these cell types, BIT has also shown inhibition of epithelial–mesenchymal transition, causing a positive up-regulation of epithelial markers such as E-cadherin and occludin, and a concomitant decrease in the protein level of mesenchymal markers, arresting cancer progression to its invasive state [91]. Likewise, BIT has caused the death of breast cancer cells, including MDA-MB-231, MCF-7, MDA-MB-468, BT-474, and BRI-JM04, through induction of autophagy, associated with an increase in FoxO1 expression and acetylation [92]. Although both MoAE and BIT have shown effectiveness against breast cancer cells in vitro, in this study, the administration of MoAE at a dose of 500 mg/kg demonstrated superior in vivo effectiveness compared to BIT at a dose of 20 mg/kg. This is evidenced in Table 4, where MoAE at 500 mg/kg resulted in a 44% reduction in the average number of tumors per group and a 70.14% decrease in cumulative tumor weight (in comparison to the DMBA group), while BIT at 20 mg/kg reduced these parameters by 33% and 67.56%, respectively.

Other compounds identified in this study in the aqueous extract of *Moringa oleifera* (MoAE), such as kaempferol, vitexin, pinolenic acid, and ramelteon (TAK-375), have also shown anticancer activity. Several preclinical investigations have highlighted the role of kaempferol in the prevention and treatment of breast cancer [93], and it has been shown to suppress the proliferation of triple negative breast cancer (TNBC) MDA-MB-231 cells by inducing G₂/M phase arrest and apoptosis [94]. Vitexin has been shown to increase apoptosis in MCF-7 cells, generating up-regulation of microRNAs, including the expression of caspase-3, -6, and -8 genes, as well as down-regulation of others [95]. Pinolenic acid inhibits cell metastasis by suppressing invasiveness and cell motility in MDA-MB-231 human breast cancer cells [96], while ramelteon, a melatonin receptor agonist, significantly suppresses endometrial cancer cell proliferation (HHUA), inhibiting invasion and reducing the expression of the MMP-2 and MMP-9 genes [97]. In addition, it reduces the incidence and intensity of postoperative delirium in elderly patients undergoing lung cancer surgery [98].

**Figure 4:**
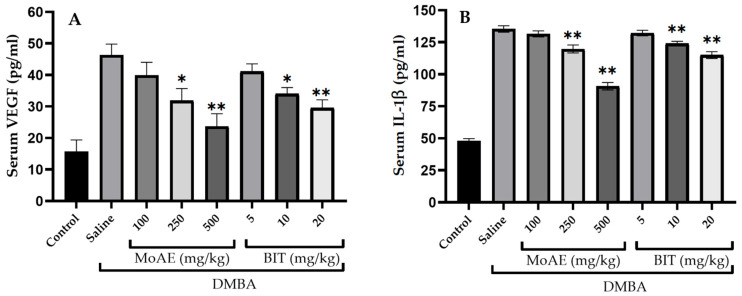
Serum levels of (**A**) vascular endothelial growth factor (VEGF) and (**B**) interleukin-1 beta (IL-1β) in rats at the end of MoAE and BIT treatment for 13 weeks. * *p* < 0.05, ** *p* < 0.001.

In the group treated only with DMBA and receiving saline as treatment, a significant increase in serum VEGF levels was observed, from 15.67 ± 3.67 pg/mL (healthy control group) to 46.32 ± 3.51 pg/mL (*p* < 0.001). Administration of MoAE at 250 mg/kg or BIT at 10 mg/kg reduced these values to 31.96 ± 3.78 and 34.06 ± 2.10 pg/mL, respectively (*p* < 0.05). At higher doses, such as 500 mg/kg MoAE and 20 mg/kg BIT, a more pronounced effect was observed, with a *p*-value < 0.001 (Figure 4A). The pattern for serum IL-1β levels was similar, with a *p*-value < 0.001 (Figure 4B). VEGF is known as an angiogenic factor, and IL-1β also has the ability to increase the formation of new blood vessels in tumors, contributing to increased tumor development and metastatic spread [99]. In this context, the results of this study suggest that the mechanism of action against breast cancer could be related, at least in part, to the down-regulation of VEGF and IL-1β. The use of pharmacological inhibitors targeting IL-1β has been suggested as a promising option to address metastasis in breast cancer [100].

## 3. Materials and Methods 

### 3.1. Plant Sample Preparation

The leaves of *Moringa oleifera* Lam were purchased from the medicinal plant market in the city of Lima, Peru. To produce MoAE, the washed leaves were dried at a temperature of 40 °C, then ground in an electric mill. Subsequently, 500 g of the resulting powder was combined with 1000 mL of distilled water at 100 °C and stirred for 15 min. Afterward, the mixture was allowed to cool, filtered under vacuum, and concentrated using a rotary evaporator. Finally, it was dried at 40 °C, resulting in 34 g of aqueous extract (yielding an extraction rate of 6.8%), which was then refrigerated at 4 °C until use.

Benzyl isothiocyanate was purchased from Sigma-Aldrich (St. Louis, MO, USA).

### 3.2. Analysis of the Chemical Composition of MoAE by UHPLC–Q-TOF/MS

We used ultra-performance liquid chromatography coupled with a triple quadrupole time-of-flight mass spectrometer (UHPLC–Q-TOF/MS) to determine the phytochemical composition of MoAE employing MetaboScape software version 4.0 for data analysis. The experimental configuration included the following parameters: LC system (Bruker UHPLC Elute Plus), column (Avantor ACE Excel C18 AQ 150 × 2.1 mm), flow rate (0.2–0.48 mL/min flow gradient), mobile phase comprising 0.01% formic acid and 0.01% acetonitrile, LC gradient (0 min 1% B, 10 min 100% B, 12 min 100% B, 12.1 min 1% B, 14 min 1% B), run time (14 min), and injection volume (4 µL). The MS system used was Q-TOF Impact II (Bruker), employing VIP-HESI ionization in both positive and negative modes, a mass range of 20–1300 *m*/*z*, and an acquisition rate of 12 Hz MS for MS/MS dynamic 16–30 Hz. Calibration was performed through internal calibration on sodium formate, and the dry temperature was set at 250 °C. Sample preparation involved dissolving 1 mg of the crude extract in 1 mL of water:acetonitrile (50:50), followed by a 20-fold dilution in water after centrifugation for injection into the LC-QTOF system. 

### 3.3. Determination of the Total Phenolic Content in MoAE

The determination of the total phenolic content in MoAE was carried out using the Singleton method [101] with slight adaptations. Gallic acid (0.1 mg/mL) was used as standard and a calibration curve was generated with concentrations of 1, 2, 3, 3, 4, and 5 mg/mL. Samples were dissolved in 2.5 mL of methanol until concentrations of 0.1 mg/mL were reached. Subsequently, 250 µL of Folin–Ciocalteu was added to 0.5 mL of the prepared solution and stirred for 5 min. Then, 1250 µL of calcium carbonate was added and the solution was allowed to stand for 60 min before reading on a UV-VIS spectrophotometer at 760 nm. The total phenolic content was expressed as milligrams of gallic acid equivalent (GAE) per gram of dry extract.

### 3.4. Antioxidant Activity: DPPH Radical Scavenging Assay

This assay procedure was carried out following the indications provided by Umamaheswari [102], using 96-well microplates. MoAE and BIT were dissolved in methanol and dilutions of 25, 50, 100, and 200 µg/mL were prepared. In each well, 100 µL of each sample dilution was mixed with 100 µL of freshly prepared DPPH solution in methanol (0.4 mM). A control, consisting of 100 µL of methanol plus 100 µL of 0.4 mM DPPH, was included along with a blank sample containing 100 µL of the same dilutions plus 100 µL of methanol (without DPPH). The samples and control were analyzed in triplicate. The mixture was then incubated at room temperature in a dark place for 30 min. After gentle shaking, the absorbance was read at 517 nm. For the calculation of the DPPH radical scavenging capacity, the following formula was used:

Scavenging activity (%) = [(A0 − A1)/A0] × 100.

In the formula, A0 corresponds to the absorbance of the reaction control, and A1 indicates the absorbance when the sample is present, adjusted for the absorbance of the sample itself (blank). A concentration-dependent graph of percentage inhibition was constructed and the inhibitory concentration 50 (IC_50_) was calculated graphically. 

### 3.5. Animals 

Female Holtzman rats with a body weight of 140 ± 10 g were used. The animals were purchased from the National Institute of Health biotherium, housed in clean cages, and maintained in a temperature and light controlled environment (12-h light/dark cycle). They had unlimited access to drinking water throughout the experiment and were fed a standard rat diet.

The research protocol received the approval of the Ethics Committee of the Faculty of Pharmacy and Biochemistry of the Universidad Nacional Mayor de San Marcos on 31 July 2020 (certificate number 004-CE-UDI-FFB-2020). 

### 3.6. Evaluation of the Effect of MoAE and BIT on Breast Cancer

Breast cancer induction was performed following the procedure described by Wang and Shang [103] with minor adjustments. A single dose of 60 mg/kg of 7,12-dimethylbenz[a]anthracene (DMBA) was administered by orogastric gavage, diluted in olive oil. Forty-eight rats were randomly assigned to eight groups, each consisting of six individuals. The control (healthy) group was called Group I. Group II received DMBA plus saline. In addition to DMBA, Groups III, IV, and V received MoAE at 100, 250, and 500 mg/kg/day, respectively, while Groups VI, VII, and VIII received BIT at 5, 10, and 20 mg/kg/day, respectively. The treatment period covered 13 weeks. Mammary tumor latency was recorded and body weight was monitored weekly. At the end of the experiment, blood was drawn under ethyl ether anesthesia for serum determinations by ELISA. Subsequently, the animals were sacrificed with an overdose of pentobarbital. All tumors in each rat were counted and excised for volume determination and histopathological analysis.

To perform the histopathological analysis, tumor samples were preserved in 10% formalin. Subsequently, they were subjected to a series of steps to dehydrate and clarify them using acetone and the solvent xylene. Then, they were embedded in paraffin, sectioned with a microtome, placed on slides, and stained with hematoxylin and eosin. Evaluation was carried out using optical microscopy.

### 3.7. Determination of Serum Levels of VEFG and IL-1β

The concentration of vascular endothelial growth factor (VEGF) and interleukin-1β in rat serum was determined by ELISA (enzyme-linked immunosorbent assay) using kits purchased from Sigma-Aldrich, according to the manufacturer’s instructions.

### 3.8. Statistical Analysis

Results were expressed as mean accompanied by standard deviation. Statistical significance was assessed by one-way analysis of variance followed by a Tukey post hoc test. Statistical analysis was performed using SPSS version 19 software. All *p*-values < 0.05 were considered statistically significant.

## 4. Conclusions

In conclusion, under the experimental conditions established in this study, both MoAE and BIT exhibited antitumor properties in the rat model of induced breast cancer. An improvement in the macroscopic and microscopic characteristics of the tumors was observed, while serum levels of VEGF and IL-1β decreased. MoAE was shown to have a high content of total phenols and a remarkable antioxidant capacity. Chemical analysis by UPLC-MS revealed the predominant presence of flavonoids such as quercetin, vitexin, and kaempferol in MoAE. It is suggested that the chemopreventive effect could be linked, at least in part, to the presence of flavonoids and isothiocyanates in the extract.

## Figures and Tables

**Table 3:** Total phenolic content and antioxidant capacity (IC_50_).

	Total Phenol Content (mg Gallic Acid Equivalents/Gram Dry Extract)	Antioxidant Activity (IC_50_) µg/mL
MoAE	135.08 ± 0.64	66.66 ± 0.53
BIT	-	122.39 ± 3.66
Vitamin C	-	8.21 ± 0.02

Values expressed as mean ± SD.

**Table 4:** Effect of MoAE and BIT on tumor parameters in DMBA-induced mammary carcinogenesis in rats.

Parameters/Groups	DMBA	DMBA + MoAE-100	DMBA + MoAE-250	DMBA + MoAE-500	DMBA + BIT-5	DMBA + BIT-10	DMBA + BIT-20
Total number of tumors	18.00	15.00	11.00	10.00	18.00	14.00	12.00
Average number of tumors per group	3.00 ± 0.34	2.50 ± 0.26 (−17%)	1.83 ± 0.43 (−39%)	1.67 ± 0.49 (−44%)	3.00 ± 0.49 (−0%)	2.33 ± 0.43 (−22%)	2.00 ± 0.49 (−33%)
Tumor latency (days)	59.83 ± 3.97	62.50 ± 6.66	63.60 ± 5.73	67.80 ± 9.86	59.80 ± 4.49	70.80 ± 5.89	71.75 ± 5.38
Cumulative tumor weight (g)	35.2	21.71(−37.37%)	18.6 (−47.16%)	10.51 (−70.14%)	30.27 (−14.00%)	19.57 (−44.40%)	11.42 (−67.56%)

Values expressed as mean ± SD.

**Table 5:** Histological classification of mammary tumors of rats treated with MoAE and BIT.

Parameter/Group	DMBA	DMBA + MoAE-100	DMBA + MoAE-250	DMBA + MoAE-500	DMBA + BIT-5	DMBA + BIT-10	DMBA + BIT-20
Tubular differentiation	2	2	2	1	2	2	2
Nuclear pleomorphism	3	3	2	3	3	3	2
Number of mitoses	1	1	1	1	1	1	1
Sum score	6	6	5	5	6	6	5
Histologic grade	II	II	I	I	II	II	I

Histological grade according to Mod Elston and Ellis. Histopathology 1991. Grade I: 3–5, Grade II: 6–7, Grade III: 8–9. Parameter scoring: Tubular differentiation: 1 = >75%, 2 = 10–75%, 3 = <10%; nuclear pleomorphism: 2 = moderate, 3 = marked; number of mitoses: 1 = <7; 2 = 7–13.

## Data Availability

This publication contains all available data.

## References

[1] Fahad Ullah M. (2019). Breast Cancer: Current Perspectives on the Disease Status. Adv. Exp. Med. Biol..

[2] World Health Organization (2023). Breast Cancer. https://www.who.int/es/news-room/fact-sheets/detail/breast-cancer.

[3] Anjum F., Razvi N., Saeed U. (2017). Effects of Chemotherapy in Breast Cancer Patients. Nat. J. Health Sci..

[4] Ji X., Lu Y., Tian H., Meng X., Wei M., Cho W.C. (2019). Chemoresistance mechanisms of breast cancer and their countermeasures. Biomed. Pharmacother..

[5] Talib W.H., Alsayed A.R., Barakat M., Abu-Taha M.I., Mahmod A.I. (2021). Targeting Drug Chemo-Resistance in Cancer Using Natural Products. Biomedicines.

[6] Castañeda A.M., Meléndez C.M., Uribe D., Pedroza-Díaz J. (2022). Synergistic effects of natural compounds and conventional chemotherapeutic agents: Recent insights for the development of cancer treatment strategies. Heliyon.

[7] de Oliveira Júnior R.G., Christiane Adrielly A.F., da Silva Almeida J.R., Grougnet R., Thiéry V., Picot L. (2018). Sensitization of tumor cells to chemotherapy by natural products: A systematic review of preclinical data and molecular mechanisms. Fitoterapia.

[8] Subramonie S., Suryadevara N., Ganapathy B., Devi G. (2019). Molecular activities in *Moringa oleifera* Linn—Review. Int. J. Res. Pharm. Sci..

[9] Abdoun K., Alsagan A., Altahir O., Suliman G., Al-Haidary A., Alsaiady M. (2022). Cultivation and Uses of *Moringa oleifera* as Non-Conventional Feed Stuff in Livestock Production: A Review. Life.

[10] Abdull Razis A.F., Ibrahim M.D., Kntayya S.B. (2014). Health benefits of Moringa oleifera. Asian Pac. J. Cancer Prev..

[11] Rode S.B., Dadmal A., Salankar H.V. (2022). Nature’s Gold (Moringa Oleifera): Miracle Properties. Cureus.

[12] Sanganna B., Chitme H.R., Vrunda K., Jamadar M.J. (2016). Antiproliferative and antioxidant activity of leaves extracts of *Moringa oleifera*. Int. J. Curr. Pharm. Res..

[13] Pamok S., Saenphet S., Vinitketkumnuen U., Saenphet K. (2012). Antiproliferative effect of *Moringa oleifera* Lam. and Pseuderanthemum palatiferum (Nees) Radlk extracts on the colon cancer cells. J. Med. Plants Res..

[14] Khalil W.K., Ghaly I.S., Diab K.A., ELmakawy A.I. (2014). Antitumor activity of *Moringa Oleifera* leaf extract against Ehrlich solid tumor. Int. J. Pharm..

[15] Tiloke C., Phulukdaree A., Chuturgoon A.A. (2013). The antiproliferative effect of *Moringa oleifera* crude aqueous leaf extract on cancerous human alveolar epithelial cells. BMC Complement. Altern. Med..

[16] Khan F., Pandey P., Jha N.K., Jafri A., Khan I. (2020). Antiproliferative effect of *Moringa oleifera* methanolic leaf extract by downregulation of Notch signaling in DU145 prostate cancer cells. Gene Rep..

[17] Adebayo I.A., Arsad H., Samian M.R. (2017). Antiproliferative effect on breast cancer (MCF7) of *Moringa oleifera* seed extracts. Afr. J. Tradit. Complement. Altern. Med..

[18] Charoensin S. (2014). Antioxidant and anticancer activities of *Moringa oleifera* leaves. J. Med. Plants Res..

[19] Förster N., Ulrichs C., Schreiner M., Müller C.T., Mewis I. (2015). Development of a reliable extraction and quantification method for glucosinolates in *Moringa oleifera*. Food Chem..

[20] Waterman C., Cheng D.M., Rojas-Silva P., Poulev A., Dreifus J., Lila M.A., Raskin I. (2014). Stable, water extractable isothiocyanates from *Moringa oleifera* leaves attenuate inflammation in vitro. Phytochemistry.

[21] Al-Asmari A.K., Albalawi S.M., Athar M.T., Khan A.Q., Al-Shahrani H., Islam M. (2015). *Moringa oleifera* as an Anti-Cancer Agent against Breast and Colorectal Cancer Cell Lines. PLoS ONE.

[22] Aloo S.O., Ofosu F.K., Muchiri M.N., Vijayalakshmi S., Pyo C.-G., Oh D.-H. (2023). In Vitro Bioactivities of Commonly Consumed Cereal, Vegetable, and Legume Seeds as Related to Their Bioactive Components: An Untargeted Metabolomics Approach Using UHPLC–QTOF-MS. Antioxidants.

[23] Jin J., Lao J., Zhou R., He W., Qin Y., Zhong C., Xie J., Liu H., Wan D., Zhang S. (2018). Simultaneous Identification and Dynamic Analysis of Saccharides during Steam Processing of Rhizomes of *Polygonatum cyrtonema* by HPLC-QTOF-MS/MS. Molecules.

[24] Krzyżanowska-Kowalczyk J., Pecio Ł., Mołdoch J., Ludwiczuk A., Kowalczyk M. (2018). Novel Phenolic Constituents of *Pulmonaria officinalis* L. LC-MS/MS Comparison of Spring and Autumn Metabolite Profiles. Molecules.

[25] Raal A., Rusalepp A., Chiru T., Ciobanu N., Talvistu K., Shusta M., Koshovyi1 O., Püssa T. (2023). Polyphenolic Compounds and Antioxidant Activity of Sea Buckthorn (*Hippophae rhamnoides* L.). Phyton-Int. J. Exp. Bot..

[26] Di Matteo P., Bortolami M., Curulli A., Feroci M., Gullifa G., Materazzi S., Risoluti R., Petrucci R. (2023). Phytochemical Characterization of Malt Spent Grain by Tandem Mass Spectrometry also Coupled with Liquid Chromatography: Bioactive Compounds from Brewery By-Products. Front. Biosci..

[27] Yagali S. (2022). Study of vibrational spectra of zwitterionic 3-Aminobutanoic acid, as supported by DFT calculations. World J. Adv. Res. Rev..

[28] Cui Y.J., Liu P., Chen R.Y. (2002). Studies on the chemical constituents of Spatholobus suberectus Dunn. Yao Xue Xue Bao.

[29] Flores P., Hellín P., Fenoll J. (2012). Determination of organic acids in fruits and vegetables by liquid chromatography with tandem-mass spectrometry. Food Chem..

[30] Gruz J., Novák O., Strnad M. (2008). Rapid análisis of phenolic acids in beverages by UPLC–MS/MS. Food Chem..

[31] Yan X., Liu M., Guo C., Lian X., Shen Y., Liu Y., Qian Y., Zhang L., Wang W., Chen D. (2023). Analysis of Metabolic Differences in the Water Extract of Shenheling Fermented by *Lactobacillus fermentum* Based on Nontargeted Metabolomics. Fermentation.

[32] Arkhipov A., Sirdaarta J., Matthews B., Cock I.E. (2014). Metabolomic Profiling of *Kigelia africana* Extracts with Anti-Cancer Activity by High Resolution Tandem Mass Spectroscopy. Pharmacogn. Commun..

[33] Kuczkowiak U., Petereit F., Nahrstedt A. (2014). Hydroxycinnamic Acid Derivatives Obtained from a Commercial Crataegus Extract and from Authentic *Crataegus* spp.. Sci. Pharm..

[34] Budan A., Bellenot D., Freuze I., Gillmann L., Chicoteau P. (2014). Potential of extracts from *Saponaria officinalis* and *Calendula officinalis* to modulate in vitro rumen fermentation with respect to their content in saponins. Biosci. Biotechnol. Biochem..

[35] Ju W.T., Kwon O.C., Kim H.B., Sung G.B., Kim H.W., Kim Y.S. (2018). Qualitative and quantitative analysis of flavonoids from 12 species of Korean mulberry leaves. J. Food Sci. Technol..

[36] Phuwajaroanpong A., Chaniad P., Plirat W.M., Konyanee A., Septama A.W., Punsawad C. (2023). Phytochemical Analysis, Antimalarial Properties, and Acute Toxicity of Aqueous Extracts of Trisamo and Jatu-Phala-Tiga Recipes. Adv. Pharmacol. Pharm. Sci..

[37] Cai R., Li X., Li C., Zhu J., Zeng J., Li J., Tang B., Li Z., Liu S., Yan Y. (2022). Standards-Based UPLC-Q-Exactive Orbitrap MS Systematically Identifies 36 Bioactive Compounds in *Ampelopsis grossedentata* (Vine Tea). Separations.

[38] Pan C., Lü H. (2019). Preparative separation of quercetin, ombuin and kaempferide from *Gynostemma pentaphyllum* by high-speed counter current chromatography. J. Chromatogr. Sci..

[39] Manasa D.J., Chandrashekar K.R. (2019). Phenolic acid profiling in the leaves of *Tabernaemontana heyneana* wall. an endemic plant of the western Ghats using ultra-high performance liquid chromatography coupled with quadrupole-time-of-flight. Asian J. Pharm. Clin. Res..

[40] No D.S., Kim I.-H. (2013). Pinolenic acid as a new source of phyto-polyunsaturated fatty acid. Lipid Technol..

[41] Shin Y.G., Cho K.H., Kim J.M., Park M.K., Park J.H. (1999). Determination of betaine in Lycium chinense fruits by liquid chromatography-electrospray ionization mass spectrometry. J. Chromatogr..

[42] Olofsson M.A., Bylund D. (2016). Liquid Chromatography with Electrospray Ionization and Tandem Mass Spectrometry Applied in the Quantitative Analysis of Chitin-Derived Glucosamine for a Rapid Estimation of Fungal Biomass in Soil. Int. J. Anal. Chem..

[43] Pastorini E., Rotini R., Guardigli M., Vecchiotti S., Persiani S., Trisolino G., Antonioli D., Rovati L.C., Roda A. (2009). Development and validation of a HPLC-ES-MS/MS method for the determination of glucosamine in human synovial fluid. J. Pharm. Biomed. Anal..

[44] Perrone D., Donangelo C.M., Farah A. (2008). Fast simultaneous análisis of caffeine, trigonelline, nicotinic acid and sucrose in coffee by liquid chromatography–mass spectrometry. Food Chem..

[45] El-Wahed A.A., Rashwan E.H., AlAjmi M.F., Khalifa S.A., Saeed A., Zhao C., Naggar Y.A., Guo Z., Musharraf S.G., Wang K. (2023). Sidr Honeys Physical and Chemical Characterization, a Comprehensive Approach through LC-MS/MS, NMR, and GC-MS Analysis. Separations.

[46] Hu W., Pan X., Abbas H.M.K., Li F., Dong W. (2017). Metabolites contributing to *Rhizoctonia solani* AG-1-IA maturation and sclerotial differentiation revealed by UPLC-QTOF-MS metabolomics. PLoS ONE.

[47] Lang R., Lang T., Bader M., Beusch A., Schlagbauer V., Hofmann T. (2017). High-Throughput Quantitation of Proline Betaine in Foods and Suitability as a Valid Biomarker for Citrus Consumption. J. Agric. Food Chem..

[48] Wu Q., Yan Q., Jiang L., Chen C., Huang X., Zhu X., Zhou T., Chen J., Yan J., Wen F. (2023). Metabolomics analysis reveals metabolite changes during freeze-drying and oven-drying of *Angelica dahurica*. Sci. Rep..

[49] Zeng J., Shi D., Chen Y., Bao X., Zong Y. (2023). *FvbHLH1* Regulates the Accumulation of Phenolic Compounds in the Yellow Cap of *Flammulina velutipes*. J. Fungi.

[50] Hazrati H., Kudsk P., Ding L., Uthe H., Fomsgaard I.S. (2022). Integrated LC-MS and GC-MS-Based Metabolomics Reveal the Effects of Plant Competition on the Rye Metabolome. J. Agric. Food Chem..

[51] Cai M., Liu Y. (2008). Quantification of salsolinol enantiomers by stable isotope dilution liquid chromatography with tandem mass spectrometric detection. Rapid Commun. Mass Spectrom..

[52] Duan Y., Petzold M., Saleem-Batcha R., Teufel R. (2020). Bacterial Tropone Natural Products and Derivatives: Overview of their Biosynthesis, Bioactivities, Ecological Role and Biotechnological Potential. Chembiochem.

[53] Plaza-Cazón J., González E., Donati E.R. (2021). *Parastrephia quadrangularis*: A Possible Alternative to Inhibit the Microbial Effect on the Generation of Acid Mine Drainage. Mine Water Environ..

[54] Hayakawa Y., Suita K., Ohnuki Y., Mototani Y., Ishikawa M., Ito A., Nariyama M., Morii A., Kiyomoto K., Tsunoda M. (2022). Vidarabine, an anti-herpes agent, prevents occlusal-disharmony-induced cardiac dysfunction in mice. J. Physiol. Sci..

[55] Miyamoto H., Yaguchi T., Ohta K., Nagai K., Nagata T., Yamamoto S., Nishizaki T. (2003). 2-pyrrolidinone induces a long-lasting facilitation of hippocampal synaptic transmission by enhancing alpha7 ACh receptor responses via a PKC pathway. Brain Res. Mol. Brain Res..

[56] Mohammed B.S., Sanadelaslam E., Salwa IA E., Ahmed S.J. (2024). HPLC-PDA-MS Identification of Phenolic Profile and in vitro Antioxidant Activity of *Adansonia digitata* L. Leaves from Sudan. Moroc. J. Chem..

[57] Osiecka D., Vakh C., Makoś-Chełstowska P., Kubica P. (2024). Plant-based meat substitute analysis using microextraction with deep eutectic solvent followed by LC-MS/MS to determine acrylamide, 5-hydroxymethylfurfural and furaneol. Anal. Bioanal. Chem..

[58] Pathan S.A., Phulpoto M.H., Solangi A.R., Khanzada A.W. (2003). Solvent dependent ^13^C NMR Chemical shifts in 6-Methyquinoline and 8-Methyquinoline. J.-Chem. Soc. Pak..

[59] Hroboňová K., Sádecká J., Čižmárik J. (2018). HPLC separation and determination of dicoumarol and other simple coumarins in sweet clover. Nova Biotechnol. Chim..

[60] Wu Q.X., Chen J., Shi Y.P. (2010). RPHPLC and NMR Study of Antioxidant Flavonoids in Extract from *Gentiana piasezkii*. J. Anal. Chem..

[61] Bojilov D., Manolov S., Ahmed S., Dagnon S., Ivanov I., Marc G., Oniga S., Oniga O., Nedialkov P., Mollova S. (2023). HPLC Analysis and In Vitro and In Silico Evaluation of the Biological Activity of Polyphenolic Components Separated with Solvents of Various Polarities from *Helichrysum italicum*. Molecules.

[62] Nour A.H., Idris A.A., Ishag O.A., Nour A.H. (2022). Chemical Composition and Repellent Activity of Methyl Cinnamate-Rich Basil (*Ocimum basilicum*) Essential Oil. J. Turk. Chem. Soc. Sect. A Chem..

[63] Cho S., Lee D.J., Jung Y.-S., Kim H.B., Cho E.J., Lee S. (2016). Phytochemical Identification from *Boehmeria nivea* Leaves and Analysis of (–)-Loliolide by HPLC. Nat. Prod. Sci..

[64] Cosme F., Vilela A., Moreira L., Moura C., Enríquez JA P., Filipe-Ribeiro L., Nunes F.M. (2020). Terroir Effect on the Phenolic Composition and Chromatic Characteristics of Mencía/Jaen Monovarietal Wines: Bierzo D.O. (Spain) and Dão D.O. (Portugal). Molecules.

[65] Sowa I., Paduch R., Mołdoch J., Szczepanek D., Szkutnik J., Sowa P., Tyszczuk-Rotko K., Blicharski T., Wójciak M. (2023). Antioxidant and Cytotoxic Potential of *Carlina vulgaris* Extract and Bioactivity-Guided Isolation of Cytotoxic Components. Antioxidants.

[66] Hamid H.A., Kupan S., Yusoff M.M. (2017). Dihydroactinidiolide from thermal degradation of β-carotene. Int. J. Food Prop..

[67] Nadaf N.H., Parulekar R.S., Patil R.S., Gade T.K., Momin A.A., Waghmare S.R., Dhanavade M.J., Arvindekar A.U., Sonawane K.D. (2018). Biofilm inhibition mechanism from extract of *Hymenocallis littoralis* leaves. J. Ethnopharmacol..

[68] Clementino L.C., Torres F.A., Velasquez A.M., Villela L., Mutue T.F., Colepicolo P., Graminha M.A. (2020). Bioguided study of the Antarctic alga *Himantothallus grandifolius* (A. Geep & E.S. Geep) indicates 13E-Docosenamide as potential antileishmanial agent. J. Appl. Pharm. Sci..

[69] Manguro L.O., Lemmen P. (2007). Phenolics of *Moringa oleifera* leaves. Nat. Prod. Res..

[70] Peñalver R., Martínez-Zamora L., Lorenzo J.M., Ros G., Nieto G. (2022). Nutritional and Antioxidant Properties of *Moringa oleifera* Leaves in Functional Foods. Foods.

[71] Fitriana W.D., Ersam T., Shimizu K., Fatmawati S. (2016). Antioxidant Activity of *Moringa oleifera* Extracts. Indones. J. Chem..

[72] Tao L., Gu F., Liu Y., Yang M., Wu X.Z., Sheng J., Tian Y. (2022). Preparation of antioxidant peptides from *Moringa oleifera* leaves and their protection against oxidative damage in HepG2 cells. Front. Nutr..

[73] Prasad S., Gupta S.C., Tyagi A.K. (2017). Reactive oxygen species (ROS) and cancer: Role of antioxidative nutraceuticals. Cancer Lett..

[74] Lambring C.B., Chen L., Nelson C., Stevens A., Bratcher W., Basha R. (2023). Oxidative Stress and Cancer: Harnessing the Therapeutic Potential of Curcumin and Analogues Against Cancer. Eur. J. Biol..

[75] Puente-Cobacho B., Varela-López A., Quiles J.L., Vera-Ramirez L. (2023). Involvement of redox signalling in tumour cell dormancy and metastasis. Cancer Metastasis Rev..

[76] Chimento A., De Luca A., D’Amico M., De Amicis F., Pezzi V. (2023). The Involvement of Natural Polyphenols in Molecular Mechanisms Inducing Apoptosis in Tumor Cells: A Promising Adjuvant in Cancer Therapy. Int. J. Mol. Sci..

[77] Vera-Ramirez L., Sanchez-Rovira P., Ramirez-Tortosa M.C., Ramirez-Tortosa C.L., Granados-Principal S., Lorente J.A., Quiles J.L. (2011). Free radicals in breast carcinogenesis, breast cancer progression and cancer stem cells. Biological bases to develop oxidative-based therapies. Crit. Rev. Oncol./Hematol..

[78] Xu D., Hu M.J., Wang Y.Q., Cui Y.L. (2019). Antioxidant Activities of Quercetin and Its Complexes for Medicinal Application. Molecules.

[79] Song X., Wang Y., Gao L. (2020). Mechanism of antioxidant properties of quercetin and quercetin-DNA complex. J. Mol. Model..

[80] Zhang Q., Pan J., Liu H., Jiao Z. (2023). Characterization of the Synergistic Antioxidant Activity of Epigallocatechin Gallate (EGCG) and Kaempferol. Molecules.

[81] Selvakumar P., Badgeley A., Murphy P., Anwar H., Sharma U., Lawrence K., Lakshmikuttyamma A. (2020). Flavonoids and Other Polyphenols Act as Epigenetic Modifiers in Breast Cancer. Nutrients.

[82] Hazafa A., Rehman K.U., Jahan N., Jabeen Z. (2020). The Role of Polyphenol (Flavonoids) Compounds in the Treatment of Cancer Cells. Nutr. Cancer.

[83] Kasiri N., Rahmati M., Ahmadi L., Eskandari N., Motedayyen H. (2020). Therapeutic potential of quercetin on human breast cancer in different dimensions. Inflammopharmacology.

[84] Noolu B., Gogulothu R., Bhat M. (2016). In Vivo Inhibition of Proteasome Activity and Tumour Growth by *Murraya koenigii* Leaf Extract in Breast Cancer Xenografts and by Its Active Flavonoids in Breast Cancer Cells. Anticancer. Agents Med. Chem..

[85] Lin C.W., Hou W.C., Shen S.C. (2008). Quercetin inhibition of tumor invasion via suppressing PKC delta/ERK/AP-1-dependent matrix metalloproteinase-9 activation in breast carcinoma cells. Carcinogenesis.

[86] Hashemzaei M., Delarami Far A., Yari A., Heravi R.E., Tabrizian K., Taghdisi S.M. (2017). Anticancer and apoptosis-inducing effects of quercetin in vitro and in vivo. Oncol. Rep..

[87] Srivastava S., Somasagara R.R., Hegde M., Nishana M., Tadi S.K., Srivastava M. (2016). Quercetin, a Natural Flavonoid Interacts with DNA, Arrests Cell Cycle and Causes Tumor Regression by Activating Mitochondrial Pathway of Apoptosis. Sci. Rep..

[88] Niazvand F., Orazizadeh M., Khorsandi L., Abbaspour M., Mansouri E., Khodadadi A. (2019). Effects of Quercetin-Loaded Nanoparticles on MCF-7 Human Breast Cancer Cells. Medicina.

[89] Bolanle J. (2018). Understanding the Anti-Cancer Activities of Moringa Isothiocyanates in Breast Cancer Cells. Ph.D. Thesis.

[90] Xiao D., Vogel V., Singh S.V. (2006). Benzyl isothiocyanate-induced apoptosis in human breast cancer cells is initiated by reactive oxygen species and regulated by Bax and Bak. Mol. Cancer Ther..

[91] Sehrawat A., Singh S.V. (2011). Benzyl isothiocyanate inhibits epithelial-mesenchymal transition in cultured and xenografted human breast cancer cells. Cancer Prev. Res..

[92] Xiao D., Bommareddy A., Kim S.H., Sehrawat A., Hahm E.R., Singh S.V. (2012). Benzyl isothiocyanate causes FoxO1-mediated autophagic death in human breast cancer cells. PLoS ONE.

[93] Wang X., Yang Y., An Y., Fang G. (2019). The mechanism of anticancer action and potential clinical use of kaempferol in the treatment of breast cancer. Biomed. Pharmacother..

[94] Zhu L., Xue L. (2019). Kaempferol Suppresses Proliferation and Induces Cell Cycle Arrest, Apoptosis, and DNA Damage in Breast Cancer Cells. Oncol. Res..

[95] Najafipour R., Momeni A.M., Mirmazloomi Y., Moghbelinejad S. (2022). Vitexin Induces Apoptosis in MCF-7 Breast Cancer Cells through the Regulation of Specific miRNAs Expression. Int. J. Mol. Cell Med..

[96] Chen S.J., Hsu C.P., Li C.W., Lu J.H., Chuang L.T. (2011). Pinolenic acid inhibits human breast cancer MDA-MB-231 cell metastasis in vitro. Food Chem..

[97] Osanai K., Kobayashi Y., Otsu M., Izawa T., Sakai K., Iwashita M. (2017). Ramelteon, a selective MT1/MT2 receptor agonist, suppresses the proliferation and invasiveness of endometrial cancer cells. Hum. Cell..

[98] Miyata R., Omasa M., Fujimoto R., Ishikawa H., Aoki M. (2017). Efficacy of Ramelteon for delirium after lung cancer surgery. Interact. Cardiovasc. Thorac. Surg..

[99] Malkova A.M., Gubal A.R., Petrova A.L. (2023). Pathogenetic role and clinical significance of interleukin-1β in cancer. Immunology.

[100] Tulotta C., Lefley D.V., Freeman K. (2019). Endogenous Production of IL1B by Breast Cancer Cells Drives Metastasis and Colonization of the Bone Microenvironment. Clin. Cancer Res..

[101] Singleton V.L., Orthofer R., Lamuela-Raventós R.M. (1999). Analysis of total phenols and other oxidation substrates and antioxidants by means of folin-ciocalteu reagent. Methods Enzymol..

[102] Umamaheswari M., Chatterjee T. (2008). In vitro antioxidant activities of the fractions of *Coccinia grandis* L. Leaf extract. Afr. J. Tradit. Complement. Altern. Med..

[103] Wang Z., Zhang X. (2017). Chemopreventive Activity of Honokiol against 7, 12-Dimethylbenz[a] anthracene-Induced Mammary Cancer in Female Sprague Dawley Rats. Front. Pharmacol..

